# Trends in compassionate use of medicinal products: Israel 2020–2024

**DOI:** 10.1186/s13584-025-00730-3

**Published:** 2025-11-13

**Authors:** Eyal Schwartzberg, Eli Marom, Victoria Finkel-Pekarsky, Segev Shani, Miriam Cohen Kandli, Mohammed Aboukaoud

**Affiliations:** 1 Clinical Pharmacy and Regulatory Management MSc. Program, School of Pharmacy, Health Sciences Faculty, Ben Gurion University, Beer Sheva, Israel; 2 Pharmaceutical Society of Israel, Tel Aviv, Israel; 3 Pharmaceutical Division, Ministry of Health, Jerusalem, Israel; 4 Import of Medicinal Products and Drugs Department, Pharmaceutical Division, Ministry of Health, Jerusalem, Israel; 5 Department of Health Management and Policy, Guilford Glazer Faculty of Business and Management and Health Sciences Faculty, Senior Lecturer, Ben Gurion University, Beer Sheva, Israel; 6 Pharmaceutical Division, Ministry of Health, Jerusalem, Israel; 7 School of Pharmacy, Health Sciences Faculty, Ben Gurion University, Beer Sheva, Israel

**Keywords:** Compassionate use, Expanded access, Israel, Drug utilization, Health policy

## Abstract

**Background:**

Compassionate use programs allow patients with life-threating and serious conditions to access investigational therapies when standard treatments are inadequate. This study aims to analyze the trends and outcomes of the compassionate use of medicinal products in Israel.

**Methods:**

Data from the Israeli Ministry of Health’s compassionate use database (2020–2024) were anonymized and analyzed. Duplicates were removed, and a pivot table was used to assess factors such as active ingredients, indications, treatment counts, and patient demographics. Results were presented as counts and percentages, with treatments over 0.5% of the total classified as common. Statistical analyses included Student’s t-test and chi-squared test for subgroup differences (*p* < 0.05 significant). Commonly used medicinal products were cross-referenced with the MOH drug database for registration and reimbursement status.

**Results:**

A total of 3,284 compassionate treatments were administered, employing 596 distinct medicinal products to address 1,361 conditions in 2020–2024. Temporal analysis identified a peak in 2020, which accounted for 24% of total treatments, followed by a decrease thereafter. Patient age stratification indicated that those aged 65 to 80 received the highest treatment proportion (26%), while the 45 to 65 age group accounted for 19%. Treatments were mainly concentrated in large central hospitals (77%) and the central district (49%), with the southern district showing the least usage. The authorization process was primarily for the continuation of study drug in 63% of cases. Additionally, Belantamab mafodotin and Trametinib were the most frequently utilized medicinal products, accounting for 9% and 8.6% of treatments, respectively. Disease category analysis revealed that relapsed refractory multiple myeloma, central nervous system tumors, and inflammatory bowel disease were among the top conditions treated, varying by age group. Notably, 60% of the most common technologies (13 out of 22) were subsequently included into the national health basket, typically following an extended period of compassionate use that exceeded two years.

**Conclusion:**

The study suggests that Israel’s compassionate use programs have accelerated early access to novel therapies for complex conditions and provided a bridge to the inclusion of novel medicinal products in the national health basket. Nonetheless, the study identifies a concerning downward trend in utilization alongside potential access disparities, thereby underscoring the necessity for further targeted investigations.

## Background

Compassionate use provides a pathway for patients to gain access to investigational drugs, biologics, and medical devices for life-threating and serious diseases or conditions (defined as causing significant disability) that might not otherwise be available. To date, the current professional literature provides limited information about the type and trends of specific medications given in the compassionate use pathway. Most manuscripts describe different regulatory frameworks in different countries, but no information is available publicly on the utilization patterns of specific medicinal products in compassionate use.

The purpose of this manuscript is to discuss and analyze the compassionate use of medicinal products in pattern utilization in Israel between 2020 and 2024. The exact number of such treatments is not publicly available, and to our knowledge, this is the first manuscript to describe in detail the landscape and scope of compassionate use of medicinal products internationally and in Israel. Prior to presenting and discussing the results of this study, it is imperative for the readers to understand the regulatory framework of compassionate care treatments.

## Regulatory framework for compassionate use of medicinal products in Israel

According to Section 47 A(b) of the Pharmacists Ordinance New Version 5741 − 1981 [1]. No person shall manufacture or market a medical product, nor order its use, unless it is registered in the Register of Medicinal Products and in accordance with the registration conditions. Regulation 29 of the Pharmacists Regulations (Medicinal Products) 5746 − 1986 lists the exceptions to this rule [2]. Within this framework, the conditions for “compassionate treatment” were also established.

The conditions for “Compassionate treatment” and “urgent treatment” are set in the Director General circular 30/2006 titled “Guidelines and Clarifications for Conditions of Administrative Approval for Compassionate Care Treatment” [3], and was subsequently updated in the Notice Regarding Director’s Approval According to the Pharmacists Regulations (Medicinal Products) of 1986, Part B - Individual Conditions, Section II 3(b) issued by the Ministry of Health (MOH) [4]. These allow physicians to prescribe an unapproved drug to save lives, prevent irreversible morbidity, and assist patients suffering from serious illnesses when there is no suitable therapeutic alternative. “Compassionate treatment” is defined as treatment with a medicinal product that is not approved in any country, given to a patient suffering from a life-threatening disease or a disease-causing significant disability (seriously debilitating), and who cannot be properly treated with a medicinal product registered and approved for marketing in Israel or in another country, and the medical treatment cannot be included in a clinical trial.

Such treatment requires prior approval from the MOH; however, there may be situations where “urgent treatment” is to be provided (urgent treatment as defined as an immediate emergency treatment to prevent death or significant disability). This treatment is with a medicinal product unapproved in any country for a patient in immediate life-threatening danger or immediate risk of organ loss or irreversible disability who cannot be properly treated with a medical preparation registered and approved for marketing in Israel or another country. Due to the immediate need, there is no time to obtain approval from the MOH for the treatment prior to its use.

Compassionate treatment must be dispensed and administered only after approval by the institutional ethics committee (“Helsinki Committee”), and the product must be used only in the hospital. The authorities approving the treatment will give it only after they are satisfied that the benefit of using the preparation outweighs the risk involved.

The treatment must be accompanied by the following documents: the patient’s medical history, diagnosis, disease state, response to previous treatment methods, requested treatment plan including monitoring methods, expected side effects and toxicity from the product, and informed consent of the patient. The request shall be accompanied by scientific rationales and evidence from professional literature, if available.

In urgent treatment, the patient’s consent may be obtained in writing shortly after providing the treatment. The treatment will be approved by the head of the Helsinki Committee of the hospital, who will then report to the Director General of the MOH about the urgent treatment given to the patient within three working days from the beginning of the treatment.

The request for compassionate treatment concerns the individual treatment of a single patient. For a larger number of patients, a clinical trial must be conducted as required. The responsibility for compassionate treatment lies with the treating physician and the medical institution where the treatment is given, even when approval from the MOH for the treatment is obtained.

At the end of the compassionate treatment, the medical institution will submit a report to the Director (Director General of the MOH) on the treatment’s results, effectiveness, and observed side effects.

Another option for receiving compassionate treatment is after the completion of a clinical trial, when the research initiator commits to continue supplying the research product, free of charge for a period of up to three years, subject to the recommendation by the site’s principal investigator that the participant’s benefit requires continued treatment with the research product, and that there is no other suitable alternative medical treatment according to the decision of the institutional ethics committee where the trial was conducted (Procedure 14 of the Pharmaceutical Division, MOH - Procedure for Clinical Trials in Human Beings, 2020 Version [5].

## Israel regulatory framework for compassionate use compared with the united States (US) and Europe

As for compassionate use, the Israeli regulatory framework is similar to what is observed in the US and Europe. The National Cancer Institute defines compassionate use as “A way to provide an investigational therapy to a patient who is not eligible to receive that therapy in a clinical trial, but who has a serious or life-threatening illness for which other treatments are not available. Compassionate use allows patients to receive promising but not yet fully studied or approved cancer therapies when no other treatment option exists, also called expanded access” [6]. The US Food and Drug Administration refers to “compassionate” use as expanded access and states that this is a potential pathway for a patient with a serious or immediately life-threatening disease or condition to gain access to an investigational medical product (drug, biologic, or medical device) for treatment outside of clinical trials when no comparable or satisfactory alternative therapy options are available [7]. The FDA takes similar steps to Israel and may allow for expanded access when all the following apply:

- Patients have a serious or immediately life-threatening disease or condition.
- There is no comparable or satisfactory alternative therapy to treat the disease or condition.
- Patient enrollment in a clinical trial is not possible.
- Potential patient benefit justifies the potential risks of treatment.
- Providing the investigational medical product will not interfere with investigational trials that could support a medicinal product’s development or marketing approval for the treatment indication.

The FDA recognizes that the investigational medicinal product may not be effective in treating the condition, and its use could lead to unexpected serious side effects.

In Europe, the European Medicine Agency (EMA) defines compassionate use as the use of an unauthorized medicine outside a clinical study in individual patients under strictly controlled conditions. This helps to make medicines that are still under development available to patients. The European Medicines Agency (EMA) provides recommendations through the Committee for Medicinal Products for Human Use (CHMP), but these do not create a legal framework [8]. Member States coordinate and implement compassionate use programs, while setting their own rules and procedures.

Established by Article 83 of Regulation (EC) No 726/2004, this tool is designed to [9]:

- Facilitate and improve access to compassionate use programs by patients in the EU;
- Favor a common approach regarding the conditions of use, the conditions for distribution and the patients targeted for the compassionate use of unauthorized new medicines;
- Increase transparency between Member States in terms of treatment availability.

These programs are only put in place if the medicine is expected to help patients with life-threatening, long-lasting, or seriously debilitating illnesses, which cannot be treated satisfactorily with any currently authorized medicine. This type of compassionate use is intended for groups of patients who have a disease and should not be confused with ‘named-patient basis’, where doctors obtain medicines directly from manufacturers before authorization. This is done on an individual basis under the direct responsibility of the doctor, and the Agency does not need to be informed.

According to EMA, medicines that are not yet authorized are first made available through clinical trials, and patients should always be considered for inclusion in trials before being offered compassionate use programs [10].

## Methods

In this retrospective cross-sectional observational study, we extracted and analyzed data from the Israeli MOH compassionate care database for 2020–2024. It should be noted that the analysis included only medicinal products and did not include advanced therapies (ATMPs), which are scarce and are managed under different procedure. To maintain data privacy, MOH personnel removed all personal patient details prior to data extraction. The MOH determined that the study was exempt from ethics committee approval as it utilized anonymized, retrospective data.

The dataset was imported into Microsoft Excel for initial processing, which included the removal of duplicates and the exclusion of incomplete records. Subsequently, a comprehensive pivot table analysis was performed examining the following parameters: Active ingredient/drug name, indication per active ingredient, number of treatments per active ingredient and indication, year of compassionate use, institution where treatment was administered, rationale for compassionate care approval, patient demographics (age and gender), dosage forms, sponsor and funding source of treatment, and therapeutic area.

After retrieving the data, we counted the number of medicinal products, the diseases they address, and the overall number of compassionate treatments provided. We then calculated the percentage of compassionate treatments for each pharmaceutical based on the total number of compassionate treatments provided. Common compassionate medicinal products were defined as those with a treatment percentage of 0.5% from the total number of treatments or higher; technologies with less than 0.5% were classified as uncommon.

To identify trends in common conditions addressed through compassionate use, we analyzed the frequency of indications in compassionate treatments. When subtypes of the same condition were treated with the same pharmaceutical, we grouped these subtypes together.

To test for differences among subgroups, we used the student’s t-test for continuous variables and the chi-squared test for categorical variables. A p-value of less than 0.05 was considered as statistically significant.

Further analysis focused on the main compassionate treatments, which comprised over 0.5% of all compassionate treatments. For these categories, we cross-referenced data with the official Israeli drug database maintained by the MOH to incorporate additional regulatory and reimbursement information: Drug registration filing date, reimbursement approval date, registered indication(s), indication(s) included in the national health services basket, and therapeutic area classification [11].

This analytical approach was designed to elucidate patterns, trends, and regulatory pathways of compassionate use treatments in Israel during the study period.

## Results

The total number of reported compassionate individual treatments in Israel between 2020 and 2024 was 3,284 (Table 1). The highest percentage of compassionate treatments occurred in 2020, reaching 24%. Since then, a downward trend has been observed, with significant differences among the groups (p-value < 0.001) to 18% in 2023 and 2024. The overall number of diseases and conditions eligible for compassionate technologies was 1,361, with 596 different medicinal products available. These medicinal products included small molecules (referred to as “ibs”), antibodies (known as “mabs”), radioactive substances, chimeric antigen receptors (CAR-T), vaccines, and recombinant proteins, covering all routes of administration. Most medicinal products were produced by global companies in the US, Europe, and Australia, while 18% were manufactured locally by small Israeli companies.

Ages 65–80 are the largest patient group receiving 26% compassionate treatments, followed by ages 45–60 at 19%, and > 80 years old at 13%, with a significant difference among the groups (p-value < 0.001). 77% of compassionate medicinal products were prescribed by central primary hospitals with more than one thousand beds. About half of the compassionate use came from Israel’s central district, and 27% from Jerusalem, while the southern district had the least (p-value < 0.001).

The primary reason for authorizing compassionate use was study drug continuation post-trial at 63%, while a much smaller proportion was for compassionate programs at 3.2%. However, in the remaining 33.6%, no reason was provided.

**Table 1:** Demographic characteristics of compassionate medicinal products 2020–2024

	Count (%)
Number of medicinal products	596
Number of indications	1361
Number of treatments Total 2024 2023 2022 2021 2020	3284 576 (18) 581 (18) 633 (19) 708 (22) 786 (24)
Age groups (Years) 0–1 1–6 6–12 12–18 18–30 30–45 45–65 65–80 > 80	24 (1) 346 (11) 264 (8) 143 (4) 215 (7) 354 (11) 627 (19) 885 (26) 426 (13)
Hospital no. of beds Central medical center > 1000 Large 500–1000 Medium 100–500 Small < 100	2540 (77) 547 (17) 180 (5.5) 17 (0.5)
Hospital district Center Jerusalem North South	1594 (49) 881(27) 515 (16) 294 (9)
Reason for authorizing compassionate care (CC) treatment Trial batch continuation Compassionate program Expanded access Not provided	2060 (63) 106 (3.2) 6 (0.18) 1114 (33.6)
Israeli compassionate Medicinal Products	105 (18)

**Fig. 1:**
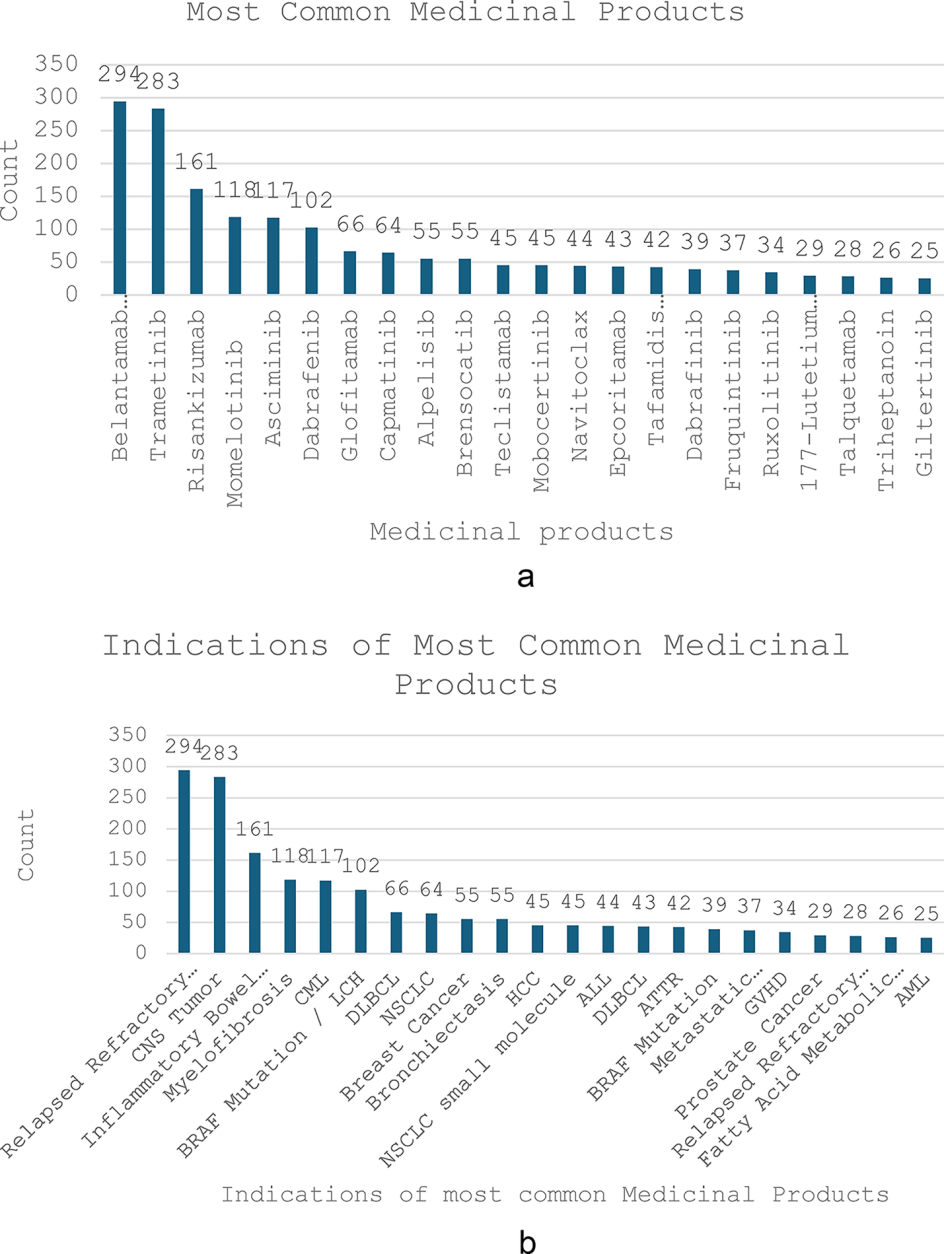
Most Common Medicinal Products for Compassionate Use (**a**) and Their Corresponding Indications (**b**) in 2020–2024

Figure 1a shows the top 22 medicinal products, which comprised 0.5% or above of the total 3284 compassionate treatments. Among the top 22 medicinal products, Belantamab mafodotin and Trametinib were the leading treatments from 2020 to 2024, accounting for 9% and 8.6% of the 3284 compassionate treatments, respectively. Notably, 64% of the top technologies depicted in Fig. 1a were small tyrosine kinase molecules, or ‘ibs’.

Figure 1b illustrates the corresponding indications for the top 22 medicinal products. Belantamab is primarily used for relapsed or refractory multiple myeloma, while Trametinib is indicated for various central nervous system (CNS) tumors, including both high-grade and low-grade gliomas, paragangliomas, neurofibromas, histiocytic tumors, and glioblastomas. In a small number of Trametinib cases, it was used to treat melanoma.

It is noteworthy that among compassionate medicinal products for relapsed refractory multiple myeloma, Belantamab was the earliest option, ranking first among the top 22 compassionate medicinal products. Later, Talquetamab was introduced as a compassionate pharmaceutical and ranked twentieth.

The third most common technology used was Risankizumab, indicated for active Crohn’s disease.

**Fig. 2:**
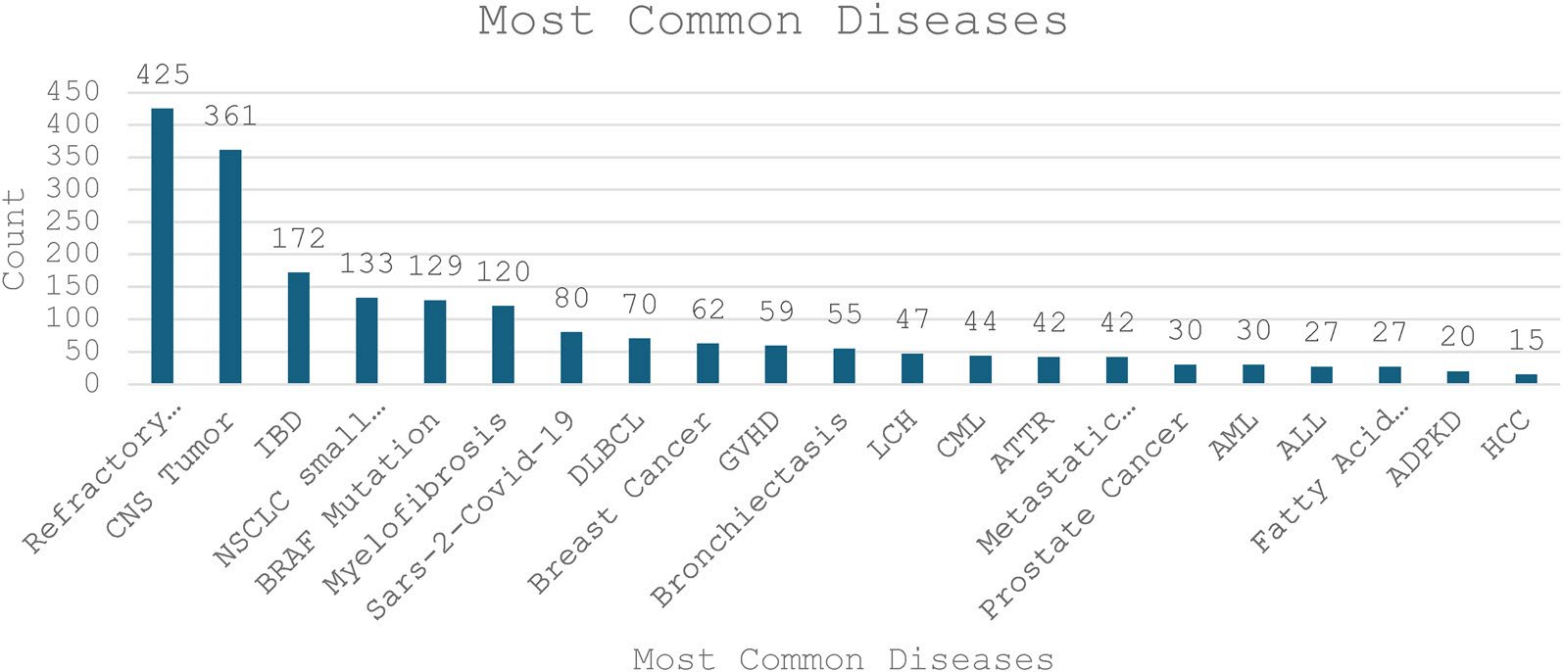
Most Common Diseases in Compassionate Use (2020–2024).central nervous system (CNS) tumors, chronic myelogenous leukemia (CML), langerhans cell histiocytosis (LCH), diffuse large b-cell lymphoma (DLBCL), non-small cell lung carcinoma (NSCLC), hepatocellular carcinoma (HCC), acute lymphoblastic leukemia (ALL), amyloid transthyretin receptor (ATTR), graft versus host disease (GVHD), acute myeloid leukemia (AML).

Figure 2 illustrates the most common diseases treated by compassionate use. The top three diseases align with the three leading technologies shown in Figure 1a: relapsed refractory multiple myeloma, CNS tumors, and inflammatory bowel disease (IBD). Non-small cell lung carcinoma (NSCLC) is notably the fourth most common type of cancer. This assessment includes all compassionate use technologies for NSCLC, which comprise 4% of total treatments. In contrast, the combined subtypes of CNS tumors exceed NSCLC, accounting for 9% of overall treatments. Although rare, fatty acid metabolic disorders are among the top 19 compassionate diseases.

We compared the leading technologies and diseases for each year. In 2020–2021, Belantamab Mafodotin was the leading technology, corresponding with the dominant disease being treated: relapsed refractory multiple myeloma. In 2022, Risankizumab emerged as the leading technology hence active Crohn’s disease became the primary disease. However, in 2023, Trametinib took the lead as the top technology, with relapsed refractory multiple myeloma again being the most treated disease. However, when considering the subtypes of CNS tumors treated by Trametinib, CNS tumors collectively surpass multiple myeloma and become the leading disease being treated in 2023. Similarly, in 2024, Momelotinib, indicated for myelofibrosis, emerged as the most common technology. While relapsed refractory multiple myeloma remained the leading treated disease, however, when myelofibrosis is grouped by subtypes, multiple myeloma is surpassed.

Table 2 illustrates the common technologies and diseases treated across different age groups. It can be inferred that up to age 18, CNS tumors are the most prevalent disease. Between the ages of 18 and 45, active Crohn’s disease emerges as the leading condition. For individuals aged 45 and older, relapsed refractory multiple myeloma becomes the primary disease being treated. Interestingly, in this age group, ATTR cardiomyopathy is identified as the second most common disease, particularly following the introduction of Tafamidis.

**Table 2:** Leading technology vs. Leading disease by age

Age group	Technology	Disease
0–1	Trametinib	• BRAF Fusion V600E Mutation Pediatric low-grade gliomas (PLGGs) • Flexiform Neurofibroma - Refractory Neurofibromatosis type 1 (NF1) • BRAF V600D Mutated Ganglioglioma
1–6	Trametinib	Refractory high-risk multisystem histiocytosis (LCH)
6–12	Trametinib	Neuroblastoma
12–18	Trametinib	• Progressive Low Grade Glioneuronal Tumor with BRAF Fusion V600E • Refractory high-risk multisystem histiocytosis (LCH)
18–30	Risankizumab	Active Crohn’s Disease
30–45	Risankizumab	Active Crohn’s Disease
45–65	Belantamab Mafodotin	Refractory multiple myeloma
65–80	Belantamab Mafodotin	Refractory multiple myeloma
> 80	Belantamab Mafodotin	Refractory multiple myeloma

Table 3 illustrates the reimbursement (Health Basket inclusion) outcomes of the top 22 medicinal products shown in Fig. 1a, which comprise about 50% of the overall compassionate treatments. The number in brackets shows the estimated percentage of the pharmaceutical out of the overall 3284 compassionate treatments. Hence, Belantamab represents 9% of the overall compassionate treatments and so on.

Most medicinal products included in the Israeli list of reimbursed healthcare services (“the basket”) are for hematology-oncology and solid tumors, including CNS tumors. Out of the most common compassionate medicinal products, 13 (60%) have been included in the basket and are reimbursed by the state of Israel. Additionally, 16 out of the 22 (72%) medicinal products that accounted for 1 ≥ 0.5% compassionate treatments were filed for registration and were approved by the MOH.

The duration of compassionate use varied between 1 and 4 years, with most medicinal products having a compassionate use period of over two years. Only one technology, Teclistamab, was added to the basket a year after the compassionate period ended. In contrast, most technologies are either still under compassionate use or were incorporated into the basket concurrently with the end of their compassionate use. Mobocertinib was withdrawn from the market but remained available for compassionate use until its withdrawal.

## Discussion

This analysis provides a comprehensive overview of actual compassionate use in Israel from 2020 to 2024, revealing significant trends in disease focus, patient demographics, geographical distribution, and reimbursement outcomes. The study identified 1,361 eligible conditions and 596 distinct medicinal products utilized within this framework, encompassing a wide range of therapeutic modalities, primarily small molecules and antibodies. While most originated from global pharmaceutical companies, a notable 18% were locally developed, highlighting a domestic capacity in specialized treatments.

Over the five-year period, 3,284 compassionate treatments were administered. A key finding is the significant downward trend in the number of treatments since the peak in 2020. Our study suggests that trial extensions are the dominant pathway for early access in this context, potential factors for this decline could include faster drug approval processes, changes in clinical trial designs, shifts in prescribing practices, or the economic impacts affecting access programs. The fact that with study drug continuation was the main authorization reason (63%), while novel agents contributed only 3.2% shows that few patients are actually receiving compassionate treatment which is not available in Israel through clinical trials.

Furthermore, since the supply of the research product to patients continues for up to three years according to MOH guidelines [5], their rapid registration and inclusion in the health basket may also contribute to the decrease in compassionate care treatments being provided. This situation may indicate that the reimbursement mechanism is efficient and enables early and regular access to new treatments for patients.

In addition, the COVID-19 pandemic could explain the surge demonstrated in our study in 2020. A study by Agarwal et al. found that there was a surge in global research and development, with the number of clinical trials increasing by 38% in 2020 due to national mobilization to address COVID-19 [12]. While this surge demonstrates national commitment to the pharmaceutical industry, it may have negative long-term implications including approval delays, industry growth slowdowns, and changes in regulatory and supply chain practices [13].

Genuine life-saving, compassionate treatments, outside of trial continuations, do occur, although often at volumes too low to appear in the top usage statistics. Examples include twenty treatments with Anti-Sars-COV-2-IVIG (Immunoglobulin antibodies) between 2021–2023 for Covid-19 in immunocompromised patients and the seventeen treatments with the anti-mold agent Fosmanogepix in 2024, reportedly used as life-saving interventions for soldiers with disseminated Fusariosis [14]. This emphasizes the social commitment of providing compassionate treatments, due to changing health circumstances as seen in the COVID pandemic and infections acquired by soldiers during the recent "Iron Swords" war in Gaza.

Demographically, compassionate use is most prevalent among older adults (65–80 years accounting for 26%), aligning with the leading diseases treated, such as relapsed refractory multiple myeloma, which dominates in patients aged 45 and older. Conversely, CNS tumors are most common in patients under 18, and active Crohn's disease leads between ages 18–45, indicating distinct unmet needs across different life stages.

Another interesting point is the heavy concentration of treatments (77%) in large medical centers (> 1000 beds) compared to medium and small-sized hospitals. On the one hand, large medical centers incorporate more specialties and experts and run most of the clinical trials in Israel. Therefore, it is logical that as the majority of compassionate care treatments are study trial treatment continuations, would be provided at these centers. However, this data indicates geographical disparity, with nearly half originating from the central district and minimal use in the south (9%), thus, pointing towards potential inequities in access linked to healthcare infrastructure and regional resource allocation.

It is interesting to observe that the trends in the diseases being tackled globally are also demonstrated in our study. Oncology, particularly hemato-oncology and CNS tumors, represents a major focus of compassionate use. Belantamab mandolin (for relapsed refractory multiple myeloma) and Trametinib (primarily for CNS tumors) in this cohort were the leading technologies overall. This finding supports the fact that Israel is part of the global network of clinical sites participating in multinational pharmaceutical companies clinical trials.

Notably, our study demonstrates the accelerating trend toward more advanced and personalized treatments for CNS tumors. In recent years, significant growth has been seen in research and clinical application of targeted therapies, moving beyond traditional surgery, radiation, and chemotherapy [15–17]. Additionally, our study demonstrates the prominence of 'ibs' (small tyrosine kinase molecules) among the top treatments, reflecting broader trends in targeted cancer therapies.

Although multiple myeloma was often the leading disease based on the top indication in several years, grouping subtypes shows the significant burden of CNS tumors. Additionally, in 2024, myelofibrosis is also being addressed significantly. Inflammatory bowel disease (specifically active Crohn's disease treated with Risankizumab) also emerged as a major therapeutic area of interest.

The pathway from compassionate use to formal reimbursement is evident, with 60% (13 out of 22) of the most common medicinal products eventually being included into the Israeli health basket (see Table 3). Most additions were for oncological indications. The typical compassionate use period exceeded two years prior to potential basket inclusion, suggesting this mechanism serves as a significant bridge for accessing novel therapies prior to formal approval and reimbursement for physicians and patients, while potentially increasing the positive reimbursement decision for these products for pharmaceutical companies providing them. However, variations do exist, with some drugs reimbursed concurrently with the end of compassionate use, one reimbursed a year later, and others remaining solely under compassionate access or being withdrawn entirely (like Mobocertinib). This highlights the dynamic nature of drug development, approval and market access.

**Table 3:** Health basket inclusion outcomes for common medicinal products presented in Fig. 1a and their proportion from the overall compassionate treatments

Drug (% of overall compassionate treatments)	Year and Indication for Basket Entry	Registered	Time in compassionate Use between 2020–2024 (Year)	Comments
Belantamab Mafodotin (9)	2022- Relapsed refractory multiple myeloma	Yes/No	2	Initially registered in 2021 and included in the basket in 2022. In 2024, the technology was withdrawn.
Trametinib (8.6)	2019-Melanoma 2022- Non-small cell lung cancer (NSCLC), Anaplastic thyroid cancer (ATC) 2023- Glioma (High and low grade), Ameloblastoma, Papillary thyroid cancer (PCT), Biliary tract cancer, Small intestine adenocarcinoma 2024- Glioma (low grade) in children >1 year	Yes	2, 3, 4	The product was included in the basket each year for various indications but remains in compassionate use for other CNS tumor indications not yet included.
Riskanizumab (4.9)	2023- Active Crohn’s disease	Yes	4	Remains in compassionate use for ulcerative colitis
Momelotinib (3.6)	2025- Myelofibrosis (MF) Intermediate or high	Yes	2	
Asciminib (3.6)	2023- Chronic myelogenous leukemia (CML)	Yes	3	
Dabrafenib (3.1)	2014- Advanced melanoma 2020- Non-Small cell lung cancer (NSCLC), Anaplastic Thyroid Cancer (ATC) 2023- High grade glioma, low grade glioma, biliary tract cancer (BTC)	Yes	3	Remains in compassionate use for other CNS tumors.
Glofitamab (2)	Not reimbursed	Yes	4	Ongoing compassionate care for Diffuse large B cell lymphoma, Mantle Cell Lymphoma (MCL)
Capmatinib (1.9)	2022- Metastatic Non-small cell lung cancer (NSCLC)	Yes	2	
Alpelisib (1.6)	2021- Breast Cancer	Yes	1	
Brensocatib (1.4)	Not incorporated	No	2	Indicated for bronchiectasis
Teclistamab (1.4)	2025- Multiple myeloma	Yes	2	In 2024 was not compassionate
Mobocertinib (1.3)	Not reimbursed	No	3	Indicated for Non-small cell lung cancer (NSCLC), withdrawn from US market in 2023
Navitoclax (1.3)	Not reimbursed	No	-	Ongoing
Epcoritamab (1.3)	2024- Diffuse Large B cell lymphoma (DLBCL) 2025- Follicular lymphoma	Yes	1	
Tafamadis (1.2)	2019- TTR-FAP, transthyretin familial amyloid polyneuropathy 2025- ATTR Cardiomyopathy	Yes	1	
Fruquintinib (1.2)	Not reimbursed	Yes	1	Metastatic Colorectal Cancer
Ruxolitinib (1)	2021- Myelofibrosis 2023- Polycythemia vera, Graft versus host disease acute or chronic (GVHD)	Yes	3	
177 Lutetium PSMA (≈ 1)	2021- Gastroenteropancreatic neuroendocrine tumors (GEP-NETs)	Yes/No (not for prostate cancer)	3	Not included for prostate cancer, the main compassionate indication in 2020–2024.
Talquetamab (≈ 1)	Not reimbursed	No	2	Relapsed Refractory Multiple Myeloma (RRMM)
Triheptanoin (≈ 1)	Not reimbursed	No	4	Fatty acid metabolic disorders
Gilteritinib (≈ 1)	2024- Acute myeloid leukemia with FLT3 mutation, FLT3 + AML	Yes	2	

Beyond evaluating clinical outcomes and access disparities, the dataset analyzed here holds significant potential utility as a source of Real-World Data (RWD). The longitudinal tracking of novel therapies administered outside of traditional clinical trials, particularly those provided through trial continuation programs, generates valuable insights into treatment patterns, effectiveness, and safety in a broader patient population than typically included in pivotal studies.

Interestingly, this study provides insights regarding medicinal products that have undergone an expedited regulatory pathway and their basket entry outcomes after a period of study drug continuation in compassionate use, as demonstrated by the case of Belantamab mofiditin.

Belantamab mafodotin was indicated for relapsed or refractory multiple myeloma, which represents a particularly difficult-to-treat condition, often considered for orphan drug development due to its high unmet medical need and limited effective therapies [18, 19]. Belantamab mafodotin, initially approved in 2020 for the treatment of relapsed refractory multiple myeloma, subsequently underwent market withdrawal due to failure to meet the phase III trial primary endpoints [20]. Yet, Belantamab mafodotin continued its use in a compassionate care setting, providing a therapeutic option for patients with relapsed or refractory multiple myeloma, simultaneously enabling several real-world studies that have focused on compassionate use or expanded access programs to capture clinical experiences with Belantamab mafodotin in a setting that reflects routine practice [21, 22]. Collectively, the available data from compassionate use programs suggest that belantamab mafodotin can continue to provide meaningful clinical activity in a highly refractory patient population [21–24].

Furthermore, the observed trends in technology adoption, such as the prevalence of tyrosine kinase inhibitors and the shifts in leading therapeutic targets year-over-year, suggest this data can serve as an early indicator of emerging changes in the therapeutic array. Systematically analyzing these trends could aid policymakers and healthcare systems in predicting and preparing for the integration of future medical innovations and forecasting associated budget impacts.

### Limitations

This analysis relies on the specific data reported for compassionate use authorizations. It doesn't delve into the clinical outcomes for patients receiving these treatments or the specific reasons behind the observed downward trend or geographical disparities. Indeed, this is an important issue as the MOH regulation requires the prescribing physician to provide the clinical outcome of the compassionate treatment. Furthermore, the categorization relies on the provided data points, and deeper granularity (e.g., specific CNS tumor subtypes beyond the examples given) might reveal further nuances. Furthermore, as market dynamics have a quick turnover, the results presented relate to a specific study time period, and analysis of different periods may reveal different trends.

### Future directions

Future researchshould prioritize investigating the root causes of both the declining trend in compassionate treatments since 2020 and the observed geographical disparities in access across Israel. Evaluating the clinical outcomes for patients receiving these therapies including treatment duration, particularly in comparison to standard care or later access post-reimbursement, would provide crucial insights into their real-world impact. Additionally, focused studies are warranted to validate the potential of using compassionate use data as a predictive tool for forecasting budget allocation for reimbursement decisions and anticipating future health basket inclusions. Finally, a deeper examination of the health basket committee's decision-making process regarding therapies previously available through compassionate use could yield valuable information for healthcare policy and planning**.**

## Conclusion

Compassionate use in Israel between 2020 and 2024 provided early access to 596 novel therapies for over 1,300 conditions, totaling in 3,284 treatments. Usage peaked in 2020 and has since declined significantly. Key therapeutic areas included oncology (multiple myeloma, various CNS tumors, NSCLC) and inflammatory bowel disease (Crohn's disease). Use was concentrated in older age groups (particularly 65–80), large central hospitals, and predominantly in the central district. Post trial study drug continuation was the main authorization driver. Importantly, a majority (60%) of the most frequently used compassionate technologies were subsequently included in the national health basket, often after a period of 2 years, underscoring the role of compassionate use as a bridge to accessing innovative treatments in Israel prior to their reimbursement. However, disparities in access and the observed downward trend warrant further attention.

## Data Availability

Data were extracted from the Israeli (MOH) compassionate care database. The data supporting this study’s findings are not openly available due to sensitivity and confidential regulatory information.
